# Structural and Mechanistic Analysis of *Drosophila melanogaster* Agmatine *N*-Acetyltransferase, an Enzyme that Catalyzes the Formation of *N*-Acetylagmatine

**DOI:** 10.1038/s41598-017-13669-6

**Published:** 2017-10-18

**Authors:** Daniel R. Dempsey, Derek A. Nichols, Matthew R. Battistini, Orville Pemberton, Santiago Rodriguez Ospina, Xiujun Zhang, Anne-Marie Carpenter, Brian G. O’Flynn, James W. Leahy, Ankush Kanwar, Eric M. Lewandowski, Yu Chen, David J. Merkler

**Affiliations:** 1 Department of Chemistry, University of South Florida, Tampa, Florida 33620 United States; 2 Department of Molecular Medicine, University of South Florida, Tampa, Florida 33612 United States; 3 Florida Center of Excellence for Drug Discovery and Innovation, 3720 Spectrum Boulevard, Suite 305, Tampa, FL 33612 United States; 4 Present Address: Johns Hopkins University, School of Medicine, Baltimore, MD 21205 USA; 5 Present Address: Moffitt Cancer Center, Tampa, FL 33612 United States; 6 Present Address: University of Florida, College of Medicine, Gainesville, FL 32610-0216 United States

## Abstract

Agmatine *N*-acetyltransferase (AgmNAT) catalyzes the formation of *N*-acetylagmatine from acetyl-CoA and agmatine. Herein, we provide evidence that *Drosophila melanogaster* AgmNAT (CG15766) catalyzes the formation of *N*-acetylagmatine using an ordered sequential mechanism; acetyl-CoA binds prior to agmatine to generate an AgmNAT•acetyl-CoA•agmatine ternary complex prior to catalysis. Additionally, we solved a crystal structure for the apo form of AgmNAT with an atomic resolution of 2.3 Å, which points towards specific amino acids that may function in catalysis or active site formation. Using the crystal structure, primary sequence alignment, pH-activity profiles, and site-directed mutagenesis, we evaluated a series of active site amino acids in order to assign their functional roles in AgmNAT. More specifically, pH-activity profiles identified at least one catalytically important, ionizable group with an apparent pK_a_ of ~7.5, which corresponds to the general base in catalysis, Glu-34. Moreover, these data led to a proposed chemical mechanism, which is consistent with the structure and our biochemical analysis of AgmNAT.

## Introduction

The discovery and characterization of enzymes involved in fatty acid amide biosynthesis has been a longstanding focus of our research^1^. One possible biosynthetic route for the fatty acid amides would be the reaction between an amine and a fatty acyl-CoA: R_1_-NH_2_ + R_2_-CO-S-CoA → R_2_-CO-NH-R_1_ + CoA-SH. Enzymes of the GCN5-related *N*-acetyltransferase family (GNAT) catalyze a similar reaction using acetyl-CoA as a substrate to generate *N*-acetylamides^2^. Acetyl-CoA-dependent *N*-acetylation by *N*-acetyltransferases is known for a diversity of amines^3–5^ in a broad range of organisms^2,5–8^. We have long suspected that enzymes identified as *N*-acetyltransferases might accept longer-chain fatty acyl-CoA thioesters as substrates or that novel *N*-acetyltransferase-like enzymes exist that utilize fatty acyl-CoA thioesters as substrates.

*Drosophila melanogaster* is an excellent model organism to study fatty acid amide biosynthesis. These insects are known to produce fatty acid amides^9,10^, its genome has been sequenced^11^, these organisms can be manipulated genetically^12^, and are inexpensive to maintain. In addition, two *N*-acetyltransferases had been identified from *D. melanogaster*, arylalkylamine *N*-acetyltransferase variant A (AANATA, also called dopamine *N*-acetyltransferase)^13^ and arylalkylamine *N*-acetyltransferase-like 2 (AANATL2)^14^. Both enzymes catalyze the *N*-acetylation of arylalkylamines, but their respective substrate specificities, kinetic mechanisms, and chemical mechanisms were not fully defined prior to our work. A search of *D. melanogaster* genome using the sequences of AANATA and AANATL2 led to the identification of six other putative arylalkylamine *N*-acetyltransferase-like enzymes, AANATL3-8^14,15^. A complete understanding of the structural and mechanistic features of these enzymes will provide tremendous insight into rules governing acyl-chain length specificity for GNAT enzymes. An exhaustive evaluation of these enzymes for different amine or acyl-CoA substrates may yield new chemistries and define a biosynthetic route to the fatty acid amides. To define the substrate specificities of these putative *N*-acyltransferases, we devised a screening strategy that involved the evaluation of a collection of amines vs. a short-chain acyl-CoA or a long-chain acyl-CoA. Our screening strategy led to the discovery that *D. melanogaster* AANATL2 will utilize dopamine, serotonin, and long-chain acyl-CoA thioesters as substrates^16,17^. These results are likely of significance to mammals because the *N*-fatty acyldopamines have been identified in the brain^18^ and the *N*-fatty acylserotonins in the gastro-intestinal tract^19^. Our application of the screening strategy to AANATL8 led to the identification of agmatine as the amine substrate with the highest (k_cat_/K_m_)_app_ for this enzyme. Thus, we have renamed AANATL8 as agmatine *N*-acetyltransferase (AgmNAT). The acetylation of agmatine points to novel agmatine related metabolites and new reactions in the degradation pathways of agmatine and arginine. A thorough study of the insect AANATs contributes to our understanding of fatty acid amide biosynthesis, enables a detailed comparison between the insect AANATs to the AANATs from other organisms, and fosters the development of insecticides targeted against insect AANATs. AANATs are suggested to be a good targets for the control of insect pests^20–23^. Furthermore, AANAT inhibitors could also lead to drugs to treat circadian rhythm disorders because serotonin *N*-acetyltransferase catalyzes the rate-determining step in melatonin biosynthesis^24^.

Agmatine, (4-aminobutyl)guanidine, was first described in 1910^25^ and was later identified as the product of arginine decarboxylation^26^. Research concerning agmatine was limited until the 1990s^27^, until the discovery that agmatine is produced in the mammalian brain^28,29^. Agmatine is distributed in many tissues, including the stomach, intestine (large and small), adrenal gland, heart, aorta, spleen, lung, vas deferens, kidney, liver, skeletal muscle, and plasma^30–32^. It is primarily located in cytoplasmic vesicles that are strongly associated with the mitochondria or endoplasmic reticulum^33,34^. Additionally, agmatine can translocate into the mitochondria^35–38^ and is likely associated with the Golgi complex, cell membrane, and nuclear membrane^39^. Agmatine is a neurotransmitter and neuromodulator in mammalian brain^27,40^, its physiological effects resulting from binding to the imidazoline (I_1_ and I_2_)^28,41,42^, α_2_-adrenergic^43^, nicotinic^44^, NMDA^45^, and serotonin receptors (5-HT2A and 5HT-3)^46^. Little is known about agmatine and its biosynthesis, degradation, elimination, and function in the fly or in insects, in general. Low levels of agmatine have been found in *D. melanogaster* ^47^ and agmatine has been reported from other insects^47–50^. Likewise, little is known about arginine decarboxylase from insects^51^. Our discovery and characterization of AgmNAT may point to unappreciated role(s) for agmatine and/or *N*-acetylagmatine in *D. melanogaster* and other insects.

Agmatine biosynthesis and degradation is shown in Supplementary Fig. S1. First, arginine decarboxylase (ADC) catalyzes the decarboxylation of arginine to generate agmatine^26–29^, followed by agmatine degradation via two main routes: (*a*) hydrolysis to urea and putrescine, as catalyzed by agmatinase (AGMAT, also known as agmatine ureohydrolase)^29,52^ or (*b*) oxidation to 4-guanidinobutanoic acid, as catalyzed by diamine oxidase (DAO) and aldehyde dehydrogenase (AlDH)^53–55^. In *Thermus thermophilus*, polyamine aminopropyltransferase (SpeE) catalyzes the formation of agmatine *N* ^1^-aminopropylagmatine^56,57^. In plants, agmatine coumaroyltransferase catalyzes the formation of *p*-coumaroylagmatine from *p*-coumaroyl-CoA and agmatine, *p*-coumaroylagmatine is thought to function in the defense system of the plant against infection^58,59^. An unexplored degradative pathway for agmatine is *N*-acetylation at the N1 position, catalyzed by AgmNAT to generate *N*-acetylagmatine – one of the subjects of this manuscript.

AgmNAT is a member of the GCN5-related *N*-acetyltransferase family (GNAT)^2^ and, in addition to the formation of *N*-acetylagmatine, this enzyme also catalyzes the production of *N*-acylpolyamines from the corresponding acyl-CoA and polyamine. We also present data showing the AgmNAT structure, substrate specificity, and kinetic and chemical mechanism for the AgmNAT-catalyzed reaction.

## Results and Discussion

### Crystal structure of AgmNAT

**Figure 1:**
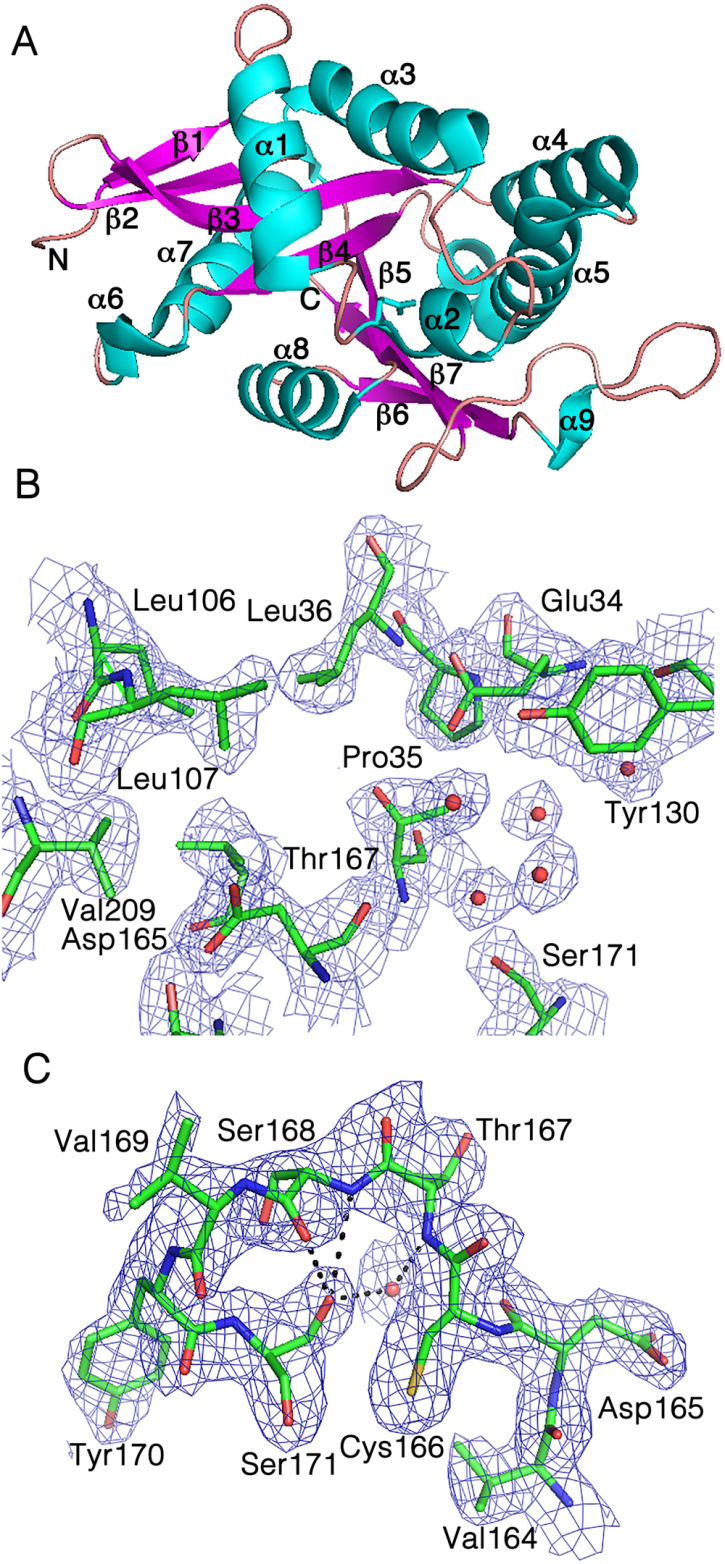
Crystal structure for AgmNAT at 2.3 Å. (**A**) Overall structure with labeled secondary structures. The two termini are labeled as N and C. (**B**) 2Fo-Fc electron density map of AgmNAT active site, contoured at 1.0 σ. Ordered water molecules are indicated by red spheres. (**C**) 2Fo-Fc electron density map (1.0 σ) of the Ser-171 region interacting with the 165–169 strand region.

**Figure 2:**
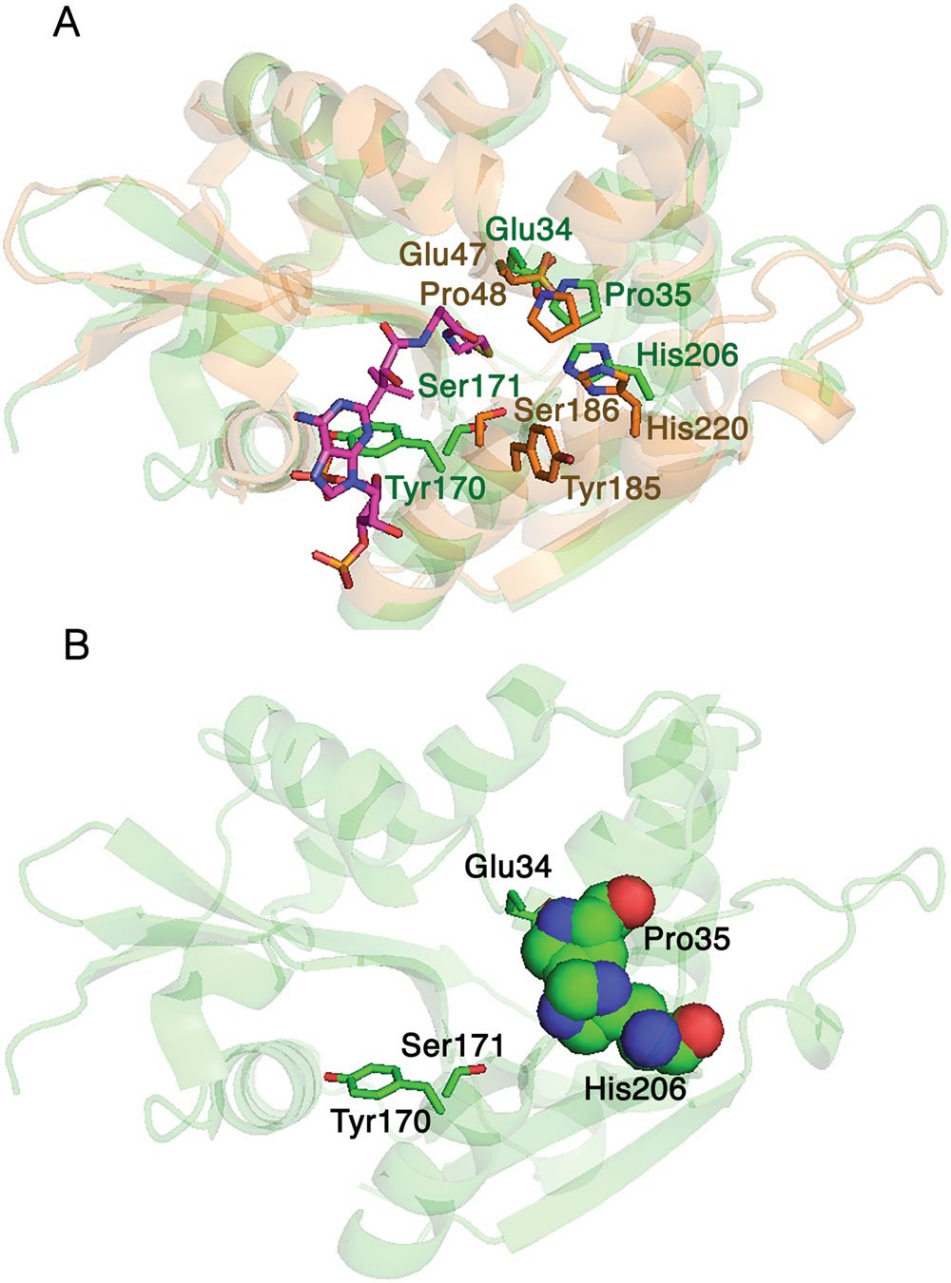
AgmNAT active site. (**A**) Superimposed structures of the apo AgmNAT (green, PDB 5K9N) and the dopamine *N*-acetyltransferase-acetyl-CoA complex (AANATA) structure (orange, PDB 3TE4) showing active site residues and acetyl-CoA (purple) from the AANATA (3TE4) structure. All relevant residues are shown in stick. The helix bearing Tyr-170 and the loop to its left form the binding site for the adenosine 3-phosphate 5-pyrophosphate portion of coenzyme (**A**). (**B**) Highlights the relative position of Glu-34, Pro-35, Tyr-170, Ser-171, and His-206 in the active site. Additionally, the van der Waals interaction between Pro-35 and His-206 is shown.

A homology model for AgmNAT was constructed using the *Aedes aegypti* arylalkylamine *N*-acetyltransferase structure^21^ as a template for molecular replacement. The AgmNAT (CG15766) crystal structure was determined at 2.3Å, with two monomers in the asymmetric unit of the P2_1_ space group (Table 1). The two monomers are nearly identical with an RMSD value of 0.262 Å when aligning 862 backbone atoms. Similar to the arylalkylamine *N*-acetyltransferase model, the new structure is primarily composed of six α-helices and seven anti-parallel α-strands (Fig. 1A). The AgmNAT structure displays a conserved GNAT fold, similar to that observed for *D. melanogaster* AANATA and human spermidine/spermine *N* ^1^-acetyltransferase (SSAT) (Supplementary Fig. S2), though the sequence identity is low when compared to these *N*-acetyltransferase enzymes (24% with AANATA and <20% for SSAT), a known feature of GNAT enzymes^2^. Based on the functional and structural similarities between AgmNAT and other GNATs such as AANATA (PDB 3TE4)^15,60^, we predict the active site pocket to be similar, though not identical, for the binding of the acyl-CoA and amine substrates (Fig. 2). The active site is well defined in the 2Fo-Fc electron density map (Fig. 1B,C) and is located near the crystal packing interface for both monomers. Based on the structure of AANATA with acetyl-CoA bound (PDB 3TE4)^60^, the binding surface for the adenosine 3-phosphate 5-pyrophosphate moiety of CoA-SH is blocked by protein-protein interactions in the AgmNAT structure, but the rest of the active site is open. The splaying of β-strand four and five, a conserved structural feature in GNAT enzymes, is also displayed in AgmNAT, which is the binding site for the pantetheine arm of acetyl-CoA^2^. Moreover, a conserved glutamate, Glu-34, that serves as the catalytic base for other *D. melanogaster N*-acyltransferase enzymes, is located within an accessible pocket that can accommodate the acyl-CoA and amine substrate, similar to that observed for AANATA (Fig. 1B)^15^. Also observed in the active site pocket are the residues, Pro-35 and Ser-171 (Fig. 1B,C), which are conserved amino acids that regulate catalysis in other *D. melanogaster N*-acyltransferases^15,61,62^. The functional roles of Pro-35 and Ser-171 of AgmNAT are discussed in subsequent sections.

**Table 1:** X-ray Data Collection and Refinement Statistics for AgmNAT.

Data Collection	
Structure	AgmNAT
Space group	P2_1_
**Cell dimensions**	
*a*, *b*, *c* (Å)	52.11
	72.35
	59.52
α, β, γ(°)	90.00
	109.64
	90.00
Resolution (Å)	50–2.1
Number of reflections	18703
R_merge_ (%)	13.9
*I*/*σI*	6.3 (*1.80)
Completeness	92.60%
Multiplicity	3.6
**Refinement**	
Resolution (Å)	50–2.3
*R*_work_/*R*_free_ (%)	20.8/28.8
**Number of heavy atoms**	
protein	3254
ligand/ion	0
water	36
***B*** **-factors (Å** ^**2**^ **)**	
Protein	28.68
ligand/ion	N/A
water	25.36
**rms deviations**	
Bond lengths (Å)	0.009
Bond angles (°)	1.27
**Ramanchandran plot**	
Most favored region (%)	96.0
Additionally allowed (%)	4.0
Generously allowed (%)	0

*Value denotes highest resolution shell (2.3 Å).

### Evaluation of acyl-CoA steady-state kinetic constants

AgmNAT showed minimal differences in the measured K_m,app_ values for acyl-CoA substrates ranging from acetyl-CoA to decanoyl-CoA (C2–C10) (Table 2) when agmatine was used as the saturating amine substrate. However, there was an acyl chain length dependent decrease in the apparent k_cat_ value for the acyl-CoA substrates as the chain length is increased. This apparent decrease in the turnover number of ~150-fold from acetyl-CoA to decanoyl-CoA, led to the observed acyl-chain length specific decrease in the (k_cat_/K_m_)_app_ value. In addition, oleoyl-CoA was not a substrate at a concentration of 500 μM. These data likely result from the acyl-chain partially (decanoyl-CoA) or fully (oleoyl-CoA) occupying the amine binding site, perturbing the productive binding of agmatine; therefore, resulting in a decrease in or complete loss of catalysis. Similar results were observed for other *D. melanogaster N*-acyltransferases^15,61,62^.

**Table 2:** Steady-state Kinetic Constants for Acyl-CoA Substrates at a Fixed Initial Concentration of Agmatine^a^ ^,b^.

Substrate (Range)^c,d^	K_m,app_ (µM)	k_cat,app_ (s^−1^)	(k_cat_/K_m_)_app_ (M^−1^s^−1^)
Acetyl-CoA (10–500 mM)	(1.0 ± 0.06) × 10^2^	23 ± 1	(2.2 ± 0.2) × 10^5^
Butyryl-CoA (1.0–750 mM)	(4.0 ± 0.6) × 10^1^	3.3 ± 0.1	(8.0 ± 1) × 10^4^
Hexanoyl-CoA (50–1000 mM)	(1.1 ± 0.03) × 10^2^	1.1 ± 0.04	(9.7 ± 0.6) × 10^3^
Octanoyl-CoA (25–1000 mM)	(1.4 ± 0.1) × 10^2^	0.61 ± 0.02	(4.4 ± 0.4) × 10^3^
Decanoyl-CoA (50–2500 mM)	(7.0 ± 1) × 10^1^	0.16 ± 0.01	(2.2 ± 0.3) × 10^3^

^a^Kinetic constants are reported as ± standard error (n = 3). ^b^Reaction conditions −300 mM Tris-HCl pH 8.5, 150 µM DTNB, 5 mM agmatine, and varying concentration of acyl-CoA. ^c^The range of acyl-CoA concentrations used in determining the K_m,app_ values at the constant, fixed initial concentration of agamatine (5 mM). ^d^Oleoyl-CoA was not accepted as substrate by AgmNAT.

### Evaluation of amine substrate steady-state kinetic constants

**Figure 3:**
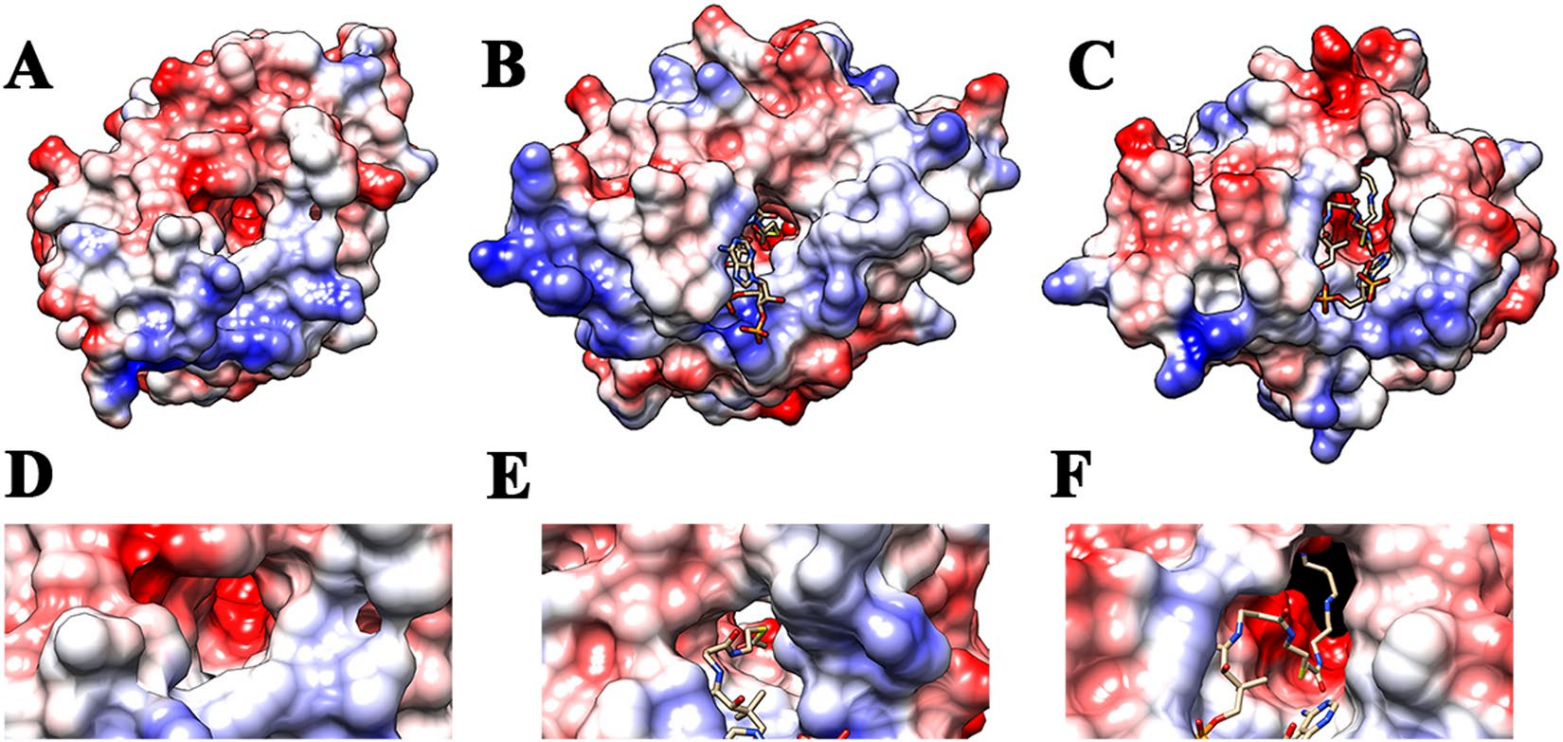
Comparison of the surface electrostatic potential of *D. melanogaster* AgmNAT to that of *D. melanogaster* AANATA and human SSAT. (**A**) AgmNAT (PDB code 5K9N). (**B**) *D. melanogaster* AANATA (PDB code 3TE4). (**C**) Human SSAT (PDB code 2JEV). (**D**) Close up of AgmNAT active site oriented to show the entry point for acetyl-CoA. (**E**) Close up of *D. melanogaster* AANATA active site with acetyl-CoA bound and oriented to show the entry point for acetyl-CoA. (**F**) Close up of the human SSAT active site with the bisubstrate inhibitor *N* ^1^-spermine-acetyl-CoA bound and oriented to show the entry point for acetyl-CoA. Blue is for positive charges and red is for negative charges. Surface electrostatic potentials reveal that the amine binding pocket for AgmNAT and SSAT are more negatively charged than the arylalkylamine binding pocket of AANATA.

We screened >50 amines as potential AgmNAT substrates using acetyl-CoA or oleoyl-CoA as the co-substrate because of our interests in fatty acid biosynthesis, structure function relationships of GNAT enzymes, and the development of novel insecticides targeted to this class of enzymes. Our amine substrate screen included the canonical amino acids (except for Cys because Cys reacts with DTNB), amino acid analogs, other biogenic amines, and different xenobiotic amines. Only six amines (Table 3) showed AgmNAT activity >3-fold higher than the level of background acetyl-CoA thioesterase activity, whereas none showed a greater rate for oleoyl-CoA. Also, we identified five polyamines as AgmNAT substrates: spermine, *N* ^8^-acetylspermidine, putrescine, spermidine, and cadaverine (Table 3). The (k_cat_/K_m_)_app_ values for the polyamines were lower than that measured for agmatine, the (k_cat_/K_m_)_app,agmatine_/(k_cat_/K_m_)_app,polyamine_ ratio ranging from 15 for spermine to 1900 for cadaverine. Structural evidence for the specificity for agmatine and different polyamines likely results from the acidic nature of the active site, similar to that observed for the human ortholog (human SSAT) (Fig. 3)^2^. A more acidic active site can accommodate an amine substrate with a basic guanidinium group better than one with a hydrophobic aromatic group, giving rise to the difference in substrate specificity when compared to an AANAT^15,60^. AgmNAT was originally named AANATL8 based on primary sequence similarity^15^; however, the substrate specificity data reported here support a new designation: agmatine *N*-acetyltransferase. This is the first report of agmatine serving as the best amine substrate for an *N*-acyltransferase. There are only a few reports of agmatine serving as a substrate within this family of enzymes^17,62,63^ and only two reports on the identification of *N*-acetylagmatine from a biological source^64,65^. Rats fed heavy-atom labeled agmatine yielded two major urinary products; heavy-atom labeled *N*-acetylagmatine and unprocessed, but labeled agmatine^64^, suggesting a similar conversion as that catalyzed by AgmNAT. Inactivation of agmatine neurotransmission by *N*-acetylation is an underappreciated reaction between arginine, agmatine, and human disease^27,66–68^, the search for a human ortholog of *Drosophila* AgmNAT could lead to a new target for drug development. Additionally, selective targeting of *Drosophila* AgmNAT could result in the development of novel insecticides for insect control^20–23^.

**Table 3:** Steady-state Kinetic Constants for Amine Substrates at a Fixed Initial Concentration of Acetyl-CoA^a^ ^,b^.

Substrate	K_m, app_ (mM)	k_cat, app_ (s^−1^)	(k_cat_/K_m_)_app_ (M^−1^s^−1^)
Agmatine (0.1–5.0 mM)	0.30 ± 0.02	18 ± 1	(6.0 ± 0.5) × 10^4^
Spermine (2.5–500 mM)	18 ± 3	77 ± 7	(4.0 ± 1) × 10^3^
*N* ^8^-acetylspermidine (0.5–50 mM)	9.1 ± 1	32 ± 2	(4.0 ± 1) × 10^3^
Putrescine (1.0–500 mM)	51 ± 3	7.7 ± 0.3	150 ± 9
Spermidine (5.0–500 mM)	17 ± 0.5	1.3 ± 0.009	76 ± 0.5
Cadaverine (2.5–1000 mM)	32 ± 6	1.0 ± 0.1	32 ± 6

^a^Kinetic constants are reported as ± standard error (n = 3). ^b^Reaction conditions −300 mM Tris-HCl pH 8.5, 150 µM DTNB, 500 μM acetyl-CoA, and varying concentration of amine substrate. ^c^he range of amine concentrations used in determining the K_m,app_ values at the constant, fixed initial concentration of acetyl-CoA (500 μM).

We found that arginine, arginine methyl ester, *N*-acetylputrescine, and *N* ^1^-acetylspermidine were not AgmNAT substrates. The ~25-fold increase in k_cat,app_ for *N* ^8^-acetylspermidine when compared to spermidine, together with our data demonstrating that *N*-acetylputrescine and *N* ^1^-acetylspermidine were not substrates all suggest that AgmNAT, most likely, catalyzes the mono- and N1- specific acetylation of these biogenic amines, similar to what is observed for the mammalian spermidine *N*-acetyltransferase^69,70^.

The increase in the k_cat,app_ value, together with the small ~2-fold difference in the K_m,app_ for *N* ^8^-acetylspermidine relative to spermidine, could result from non-productive binding of the N8-amine of spermidine in the AgmNAT active site, whereby the N1-amine is better positioned for catalysis: deprotonation and then nucleophilic attack of the –NH_2_ at the carbonyl of the acetyl-CoA thioester moiety. This means both of the amine moieties can bind in the active site, but only the N1-amine is acetylated.

While arginine and arginine methyl ester are not AgmNAT substrates, we further evaluated these for AgmNAT inhibition to determine if either could bind to the enzyme. Arginine methyl ester proved to weakly inhibit AgmNAT, decreasing the rate of *N*-acetylagmatine formation from acetyl-CoA and agmatine by ~50% at 10 mM. In contrast, we found no inhibition of *N*-acetylagmatine formation at both 10 mM and 25 mM arginine. These data show that a modification of the α-position of agmatine inhibits binding to AgmNAT and that the inhibition results from both electronic and steric effects. The presence of the negatively charged α-carboxylate seems to eliminate or significantly weaken AgmNAT binding, likely the result of charge-charge repulsion. Evidence for this suggestion comes from the weak inhibition by arginine methyl ester (K_i,s_ and K_i,i_ ≥ 1 mM, Supplementary Fig. S3), but no apparent inhibition by arginine at a concentration as high as 25 mM.

### Kinetic mechanism

**Figure 4:**
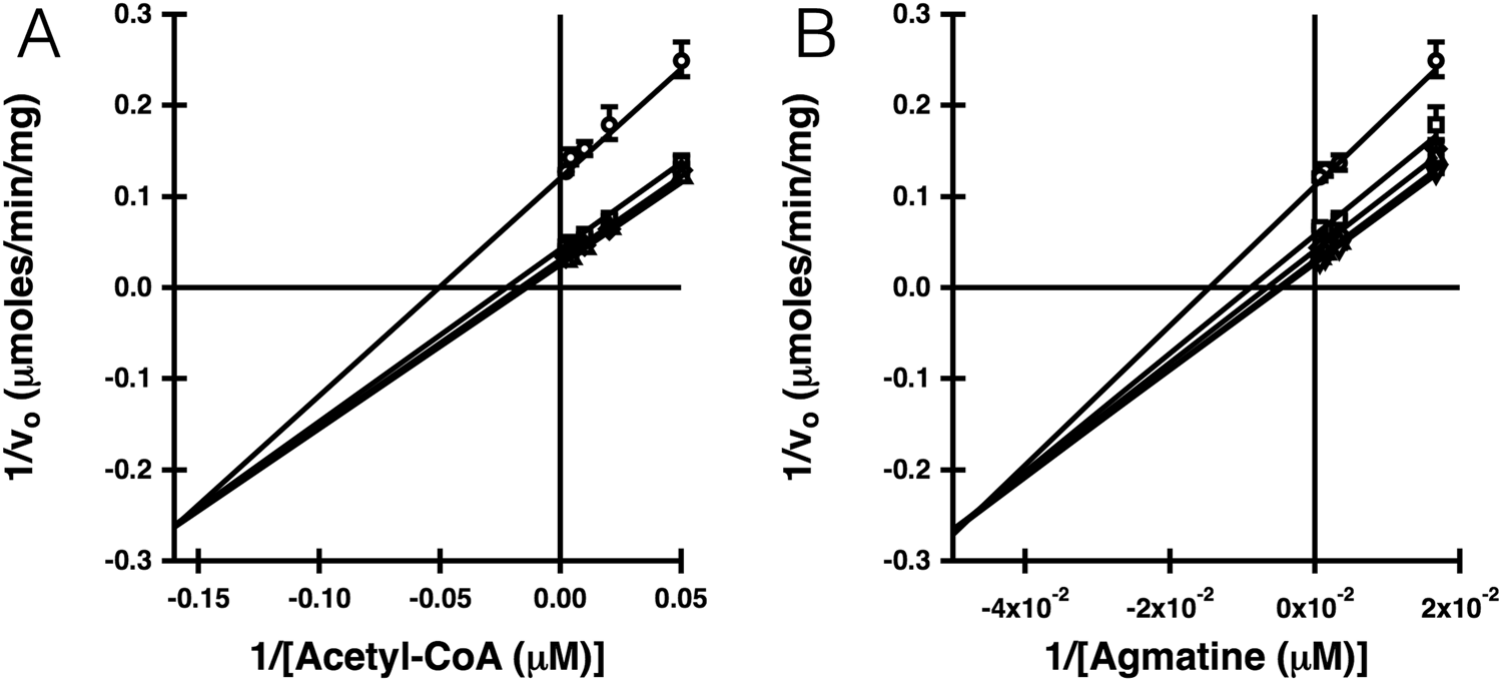
Acetyl-CoA and agmatine initial velocity double reciprocal plots. (**A**) Velocities measured at fixed concentrations of agmatine: 1500 μM (○), 750 μM (□), 300 μM (◇), and 60 μM (Δ). (**B**) Velocities measured at fixed concentrations of acetyl-CoA: 500 μM (○), 250 μM (□), 100 μM (◇), 50 μM (Δ), and 20 μM (▽). These data are best fit to the rate equation for a sequential mechanism (*χ* ^2^ is 4.6 for Equation 3) than the rate equation for a ping pong mechanism (*χ* ^2^ is 5.0 for Equation 4).

A combination of initial velocity kinetic experiments, dead-end inhibition, and product inhibition studies were used to determine the AgmNAT kinetic mechanism. Our first set of experiments was to vary the initial concentrations of one substrate at different fixed concentrations of the second substrate and fit these data to rate equations for either a sequential (Equation 3) or a ping pong (Equation 4) kinetic mechanism. Equation 3, for a sequential mechanism, provided the best fit (*χ* ^2^ is 4.6 for Equation 3 while *χ* ^2^ is 5.0 for Equation 4) which resulted in intersecting double reciprocal lines for acetyl-CoA (Fig. 4A) and agmatine (Fig. 4B). These data suggest that the AgmNAT-catalyzed formation of *N*-acetylagmatine occurs via a sequential mechanism; catalysis takes place only after formation of the AgmNAT•acetyl-CoA•agmatine ternary complex.

Next, we determined if the AgmNAT kinetic mechanism is an ordered or random sequential mechanism by using substrate analogs, oleoyl-CoA, arcaine, and arginine methyl ester, as dead-end inhibitors vs. acetyl-CoA and agmatine. The inhibitor data is summarized in Table 4 and we have included the double reciprocal plots for the inhibitors in the Supplementary Materials. Arcaine is structurally related to agmatine, with its primary amine moiety replaced with a guanidinium group. Arcaine serving as an AgmNAT inhibitor supports our conclusion that AgmNAT does not acetylate the guanidinium amine of agmatine. None of these inhibitors showed any rate of catalysis above the slow, background rate of acetyl-CoA or oleoyl-CoA hydrolysis. Oleoyl-CoA produced competitive and noncompetitive inhibition plots for acetyl-CoA and agmatine (Table 4 and Supplementary Fig. S4A,B). Arcaine produced uncompetitive and competitive inhibition plots for acetyl-CoA and agmatine (Table 4 and Supplementary Fig. S4C,D). As observed for arcaine, arginine methyl ester produced uncompetitive and competitive inhibition plots for acetyl-CoA and agmatine (Table 4 and Supplementary Fig. S3). These data demonstrate that AgmNAT catalyzes the formation of *N*-acetylagmatine through an ordered sequential mechanism: acetyl-CoA binding first followed by agmatine to generate the AgmNAT•acetyl-CoA•agmatine complex prior to catalysis. This is similar to the kinetic mechanism for other *D. melanogaster* GNAT enzymes, including AANATA, AANATL2, and AANATL7^15–17^.

**Table 4:** Inhibitor Data for AgmNAT^a^ ^,b^.

Inhibitor	Varied Substrate	Constant Substrate	Inhibitor Pattern^c^	K_i,s_	K_i,i_
Arginine methyl	Acetyl-CoA	Agmatine (0.3 mM)	UC	1.5 ± 0.1 mM	2.9 ± 0.1 mM
ester	Agmatine	Acetyl-CoA (0.1 mM)	C		
Arcaine	Acetyl-CoA	Agmatine (0.3 mM)	UN	28 ± 1 μM	34 ± 1 μM
	Agmatine	Acetyl-CoA (0.1 mM)	C		
*N*-Acetylagmatine	Acetyl-CoA	Agmatine (0.3 mM)	UC	160 ± 30 μM	420 ± 20 μM
	Agmatine	Acetyl-CoA (0.1 mM)	C		
Oleoyl-CoA	Acetyl-CoA	Agmatine (0.3 mM)	C	19 ± 1 μM	67 ± 4 μM
	Agmatine	Acetyl-CoA (0.1 mM)	NC	67 ± 4 μM	

^a^Details for each set of inhibition experiments are provided in the legends to appropriate figures included in the supplementary figures. ^b^Inhibition constants are reported as ± standard error. ^c^C = Competitive inhibition, NC = noncompetitive inhibition, and UC = uncompetitive inhibition

Support for ordered sequential mechanism for AgmNAT comes from a statistically better fit to Equation 3 (as shown in Fig. 4) and the noncompetitive inhibition of oleoyl-CoA vs. agmatine (Table 4 and Supplementary Fig. S4A,B). Additional support and further details for the kinetic mechanism are revealed by *N*-acetylagmatine product inhibition. *N*-Acetylagmatine produced uncompetitive and competitive inhibition plots for acetyl-CoA and agmatine (Table 4 and Supplementary Fig. S5). Uncompetitive inhibition by *N*-acetylagmatine vs. acetyl-CoA (Supplementary Fig. S5A) is inconsistent with a ping pong kinetic mechanism. In sum, the kinetic analyses are consistent with two kinetic mechanisms: (*a*) ordered sequential substrate binding with acetyl-CoA binding first followed by ordered sequential product release with *N*-acetylagmatine being released last or (*b*) ordered sequential substrate binding with acetyl-CoA binding first followed by ordered sequential product release with CoA-SH being being released last. Uncompetitive inhibition by *N*-acetylagmatine vs. acetyl-CoA would be explained by the formation of a non-productive AgmNAT•acetyl-CoA•*N*-acetylagmatine complex with no reversible connection between the AgmNAT•acetyl-CoA complex and the AgmNAT•CoA-SH complex. We favor the latter mechanism because we have demonstrated that CoA-SH will bind to other *D. melanogaster* AANATs^15,61^ and many other *N*-acetyltransferases exhibit ordered product release with CoA-SH being released last^71–74^.

### Proposed AgmNAT chemical mechanism

**Figure 5:**
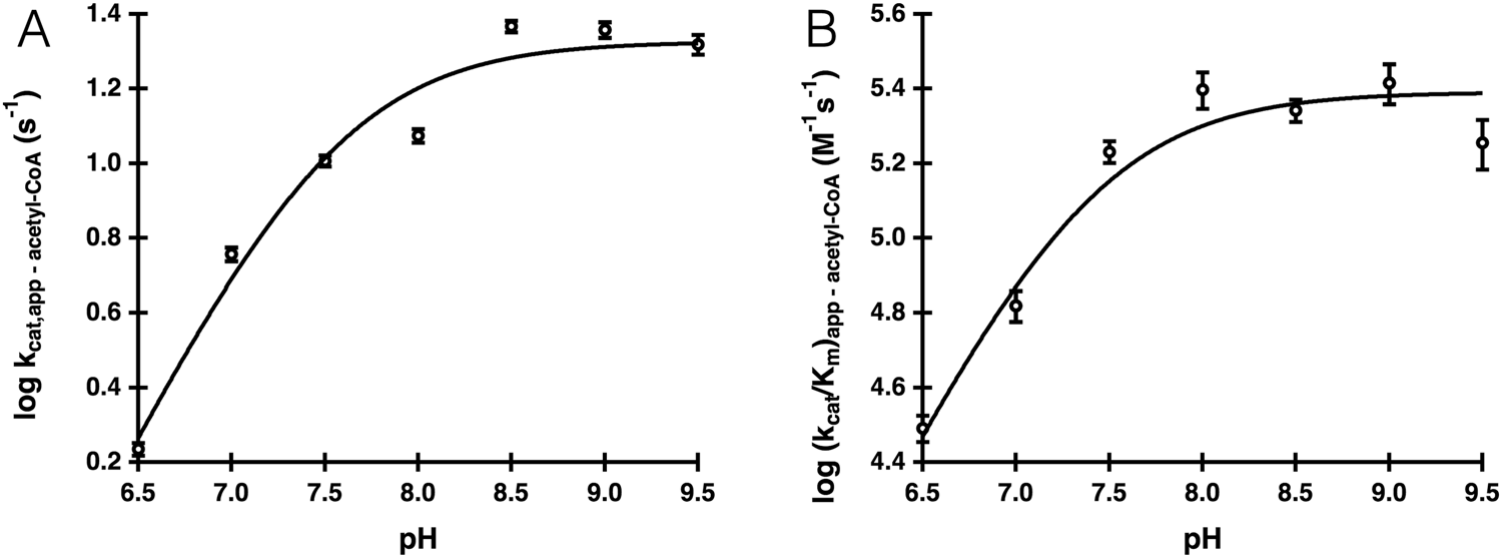
Wild-type pH-rate profiles. (**A**) k_cat,app_ for acetyl-CoA. (**B**) (k_cat_/K_m_)_app_ for acetyl-CoA.

We combined the pH-dependence of the kinetic constants, primary sequence alignment to other *D. melanogaster* GNAT enzymes^15^, determination of three-dimensional structure, and site-directed mutagenesis of a putative catalytically important residue to provide insights into the AgmNAT chemical mechanism. First, the pH-dependence of the kinetic constants was assessed for acetyl-CoA to assign apparent pK_a_ values to ionizable groups involved in catalysis. Both the k_cat,app_ and (k_cat_/K_m_)_app_ pH-rate profiles produced a rising profile with a pK_a,app_ of 7.7 ± 0.1 and 7.3 ± 0.2, respectively (Fig. 5). An apparent pK_a_ of ~7.5 can be attributed to a general base in catalysis, likely either deprotonation of the primary amine of agmatine or the zwitterionic tetrahedral intermediate generated upon nucleophilic attack of agmatine at the carbonyl thioester of acetyl-CoA. A second, higher pK_a,app_, possibly resulting from the deprotonation of a catalytically important general acid, was not observed in our pH-activity data, a surprising result given that a pK_a_ ~8.5–9.5 has been observed for many other *N*-acyltransferases^2,75,76^. Explanations for these data include: (*a*) AgmNAT catalysis does not require a general acid, (*b*) the general acid in catalysis is not rate-limiting under our assay conditions, or (*c*) the general acid in AgmNAT catalysis has an apparent pK_a_ > 9.5. Because of the high rate of base-catalyzed acyl-CoA hydrolysis, we cannot perform experiments at pH > 9.5 to define a pK_a_ > 9.5.

**Figure 6:**
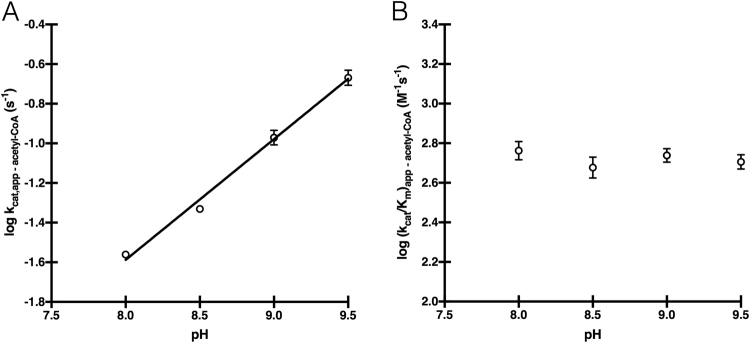
E34A pH-rate profiles. (**A**) k_cat,app_ for acetyl-CoA. (**B**) (k_cat_/K_m_)_app_ for acetyl-CoA.

Next, we combined information from primary sequence alignments, the AgmNAT structure, and site-directed mutagenesis to define potential amino acids that could function in catalysis. A conserved glutamate has been proposed as the catalytic base in two *D. melanogaster* arylalkylamine *N*-acetyltransferases (AANATs), which corresponds to Glu-34 in AgmNAT^15,16^. Additionally, the AgmNAT structure shows that Glu-34 is in the active site, a buried region with several structural waters positioned within proximity of Glu-34 (Fig. 1B), similar to *D. melanogaster* AANATA (PDB code: 3TE4)^15^. Ordered water molecules within the active site of other GNAT enzymes are thought to form a “proton wire” that assists the general base in catalysis^2,15,17,63,75–77^. Although only a number of water molecules (36 in total) were sufficiently ordered to be modeled in the current structure, the majority of them are in the active sites of the two monomers. The closest ordered water molecules to Glu-34 is ~ 3.7 Å from the Oε_1_, positioned slightly too far for a hydrogen bond; however, we anticipate that the conformational changes upon substrate binding could promote hydrogen bond interactions between ordered water molecules and the functional groups in AgmNAT and substrate. Such hydrogen bonds could facilitate proton transfer from the amine substrate to initiate catalysis. In addition, unlike Glu-33, which is exposed to the bulk solvent, Glu-34 is relatively sheltered and placed close to the hydrophobic core of the protein and next to residues such as Leu-36. This microenvironment could be responsible for a pK_a_ shift of Glu-34, as that identified in the pH-rate profiles. Therefore, we sought to interrogate the catalytic role of Glu-34 by evaluating the kinetic constants of the E34A mutant. The E34A mutation produced a catalytically deficient enzyme, exhibiting only 0.05–0.07% of the wildtype k_cat,app_ value indicating that Glu-34 does function in the catalytic cycle. Furthermore, Glu-34 seems to have a role in substrate binding because the K_m,app_ values for both agmatine and acetyl-CoA for the E34A mutant differ from wildtype values, the K_m,app_ for agmatine increases 20-fold and the K_m,app_ for acetyl-CoA decreases 6-fold (Table 5). The data generated for the E34A mutant is consistent, but does not prove, that Glu-34 serves as the general base in AgmNAT catalysis. To further investigate the role of Glu-34 in catalysis, we generated pH-activity profiles for the E34A mutant (Fig. 6). The k_cat,app_ profile produced a pH-dependent linear increase with slope of 0.7 and (k_cat_/K_m_)_app_ profile with no slope. Attempts to titrate the pH < 8.0 were unsuccessful, by which a rate of CoA-SH release was not observed above the background hydrolysis rate. The linear profile in both the k_cat,app_ and (k_cat_/K_m_)_app_ pH profiles, combined with the deficiency in catalytic rate suggest that Glu-34 serves as the general base in catalysis.

**Table 5:** Steady-state Kinetic Constants for AgmNAT Site-directed Mutants^a^.

Mutant^b^	Acetyl-CoA			
	K_m,app_ (μM)	k_cat,app_ (s^−1^)	(k_cat_/K_m_)_app_ (M^−1^s^−1^)	$\frac{{({{\boldsymbol{k}}}_{{\boldsymbol{cat}}}/{{\boldsymbol{K}}}_{{\boldsymbol{m}}})}_{{\boldsymbol{app}}\mbox{--}{\boldsymbol{mutant}}}}{{({{\boldsymbol{k}}}_{{\boldsymbol{cat}}}/{{\boldsymbol{K}}}_{{\boldsymbol{m}}})}_{{\boldsymbol{app}}\mbox{--}{\boldsymbol{wild}}\mbox{--}{\boldsymbol{type}}}}\times {\bf{100}}( \% )$
Wild-type	(1.0 ± 0.06) × 10^2^	23 ± 1	(2.2 ± 0.2) × 10^5^	100
E34A	18 ± 3	0.011 ± 0.0004	(6.0 ± 1) × 10^2^	0.27
P35A	(2.3 ± 0.3) × 10^2^	0.43 ± 0.03	(1.9 ± 0.3) × 10^3^	0.86
S171A	24 ± 1	1.84 ± 0.03	(7.5 ± 0.3) × 10^4^	34
H206A	(2.4 ± 0.3) × 10^2^	1.3 ± 0.1	(5.0 ± 1) × 10^3^	2.3
**Mutant** ^**c**^	**Agmatine**			
	**K**_**m,app**_ **(mM)**	**k**_**cat,app**_ **(s** ^**−1**^ **)**	**(k**_**cat**_ **/K**_**m**_ **)**_**app**_ **(M** ^**−1**^ **s** ^**−1**^ **)**	$\frac{{({{\boldsymbol{k}}}_{{\boldsymbol{cat}}}/{{\boldsymbol{K}}}_{{\boldsymbol{m}}})}_{{\boldsymbol{app}}\mbox{--}{\boldsymbol{mutant}}}}{{({{\boldsymbol{k}}}_{{\boldsymbol{cat}}}/{{K}}_{{m}})}_{{\boldsymbol{app}}\mbox{--}{\boldsymbol{wild}}\mbox{--}{\boldsymbol{type}}}}\times {\bf{100}}( \% )$
Wild-type	0.30 ± 0.02	18 ± 1	(6.0 ± 0.5) × 10^4^	100
E34A	6.0 ± 1	0.013 ± 0.001	2.3 ± 0.3	0.0038
P35A	0.18 ± 0.02	0.36 ± 0.02	(1.9 ± 0.2) × 10^3^	3.2
S171A	1.0 ± 0.2	1.7 ± 0.1	(1.6 ± 0.3) × 10^3^	2.7
H206A	0.42 ± 0.04	1.2 ± 0.1	(2.8 ± 0.3) × 10^3^	4.7

^a^Kinetic constants are reported as ± standard error (n = 3). ^b^Reaction conditions −300 mM Tris-HCl pH 8.5, 150 µM DTNB, varying concentration of acetyl-CoA, and saturating concentration of agmatine from 0.1–500 mM. ^c^Reaction conditions −300 mM Tris-HCl pH 8.5, 150 µM DTNB, varying concentration of agmatine, and saturating concentration of acetyl-CoA from 10–2500 μM.

**Figure 7:**
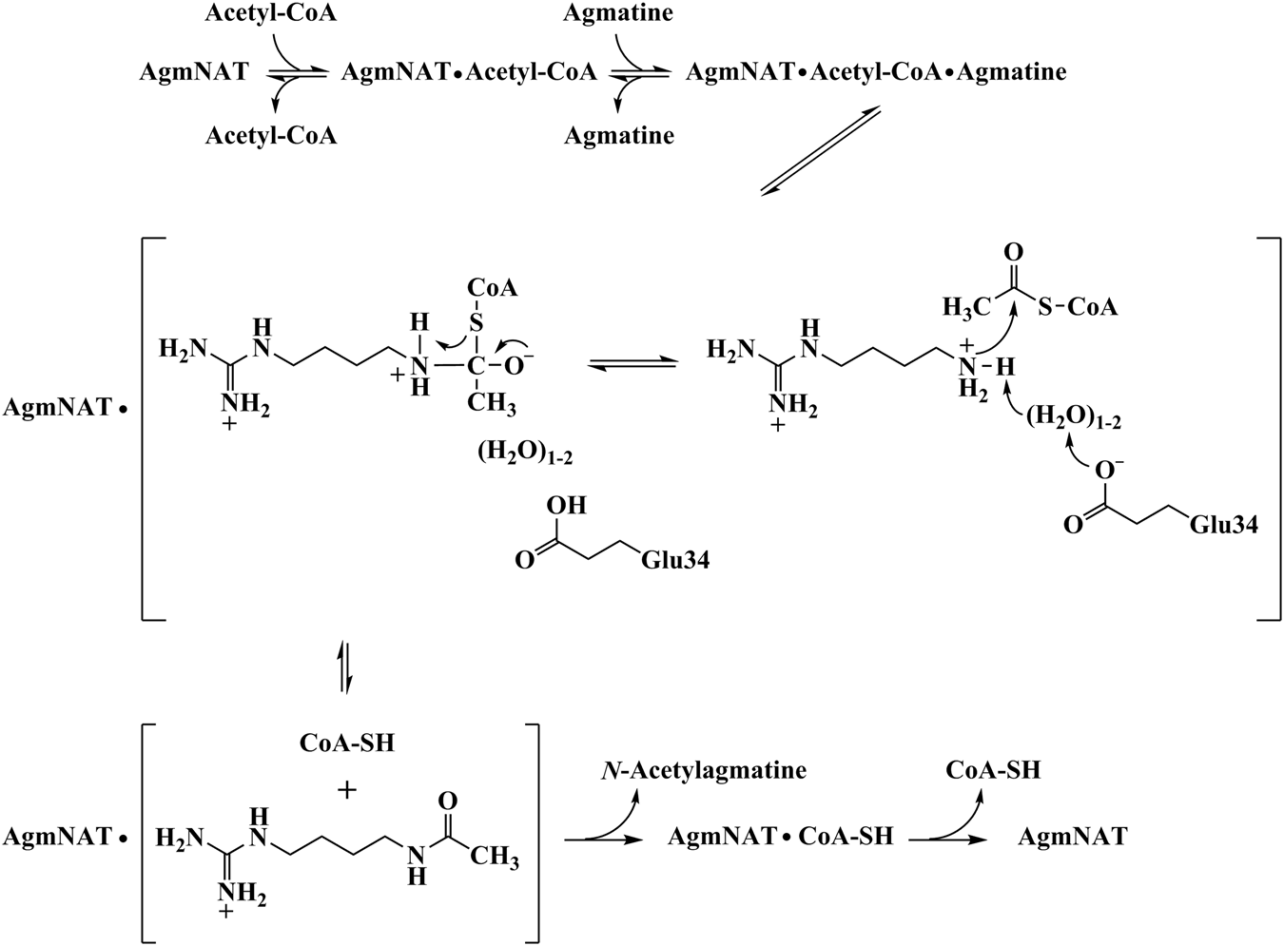
Proposed chemical mechanism for *D. melanogaster* AgmNAT.

Our steady-state kinetic data identified an ordered sequential mechanism with acetyl-CoA binding first, followed by agmatine to generate the AgmNAT•acetyl-CoA•agmatine ternary complex prior to catalysis. After the ternary complex formation, Glu-34 functions as the general base to deprotonate the positively charged amine moiety of agmatine, most likely involving a “proton wire” of ordered water molecules, followed by nucleophilic attack of the carbonyl of the acetyl-CoA thioester to generate a zwitterionic tetrahedral intermediate. Breakdown of the intermediate ensues by the departure of coenzyme A, which is, most likely, protonated by the positively charged amine of the intermediate (Fig. 7). This mechanism is consistent with other proposed chemical mechanisms for the *N*-acyltransferases of *D. melanogaster* and other organisms^15,16,24,78^.

### Other amino acids in AgmNAT that function in substrate binding and modulating catalysis

In addition to Glu-34, three other amino acids were individually mutated to alanine to define their function. These residues, Pro-35, Ser-171, and His-206, are conserved between *D. melanogaster* GNAT enzymes^15^ and are proposed to function in active site formation, substrate binding, and/or regulation of catalysis^16,17^. The P35A mutant is catalytically deficient, with a k_cat,app_ value that is ~2% of wildtype, while exhibiting only minimal K_m,app_ differences when compared to wildtype for both acetyl-CoA and agmatine (Table 5). Similar results were observed for the corresponding proline in other GNAT enzymes, except most exhibited a significant K_m_ increase for the corresponding amine, suggesting a role in substrate binding. Furthermore, the structure of sheep serotonin *N*-acetyltransferase (PDB code: 1CJW), co-crystalized with the tryptamine-acetyl-CoA bisubstrate inhibitor, shows that the corresponding Pro-64 interacts with this inhibitor via a CH-π interaction with the negatively charged face of the aromatic tryptamine moiety^77,79^. Agmatine lacks an aromatic moiety; thus, the Pro-35 of AgmNAT cannot form a CH-π interaction with agmatine, which we propose is the reason for no K_m_ effect for the P35A mutant. The decrease in k_cat,app_ for the P35A mutant indicates that Pro-35 is important for catalysis, but it seems unlikely that Pro-35 directly functions in catalysis. More likely, Pro-35 regulates active site dynamics contributing to the transition of AgmNAT from a low activity → high activity state, a conformational feature observed for other GNAT enzymes^15–17,79^. In the current AgmNAT structure, Pro-35 is stacked on top of the imidazole ring of His-206 side chain (Fig. 2). The extensive van der Waals interaction may make significant contributions to particular active site configurations.

Another active site residue evaluated for its role in substrate binding and catalysis is Ser-171. The S171A mutant only retained ~9% of the wildtype k_cat,app_ and also showed a 3- to 4-fold change in the K_m,app_ values for the substrates (a decrease in the K_m,app_ for acetyl-CoA and an increase in the K_m,app_ for agmatine) (Table 5). The decrease in the k_cat,app_ could be interpreted that Ser-171 functions as a general acid in catalysis to protonate CoA-S^-^ as it leaves the AgmNAT active site. For Ser-171 to function as a general acid during catalysis, the pK_a_ of the serine hydroxyl would have to decrease by ~3-5 pH units to protonate the thiolate anion of the CoA product. We did not observe an apparent pK_a_ in the pH-rate profiles that would correspond to a general acid, arguing against Ser-171 serving in this role. Alternatively, Ser-171 could have an important role in organizing the active site architecture to accommodate both substrates to enable efficient catalysis. Ser-171 is located in the active site, where its Oγ side chain atom forms hydrogen bonds with the backbone oxygen and nitrogen atoms of Ser-168, and a water-mediated interaction with the Thr-167 backbone nitrogen atom, suggesting that the 165–169 strand region in addition to Ser-171 is important in stabilizing the active site pocket to accommodate both substrates and allow for efficient catalysis to occur (Fig. 1C).

The H206A mutant resulted in a k_cat,app_ value that is ~18-fold lower than the wild-type value, whereas the K_m,app-acetyl-CoA_ and K_m,app-agmatine_ increased 2.3-fold and 1.4-fold, respectively. The corresponding residue (His-220) in *D. melanogaster* AANATA^15^ was shown to interact with Tyr-185 and Pro-48 to form part of the active site, an interaction potentially resulting from a conformational change driven by acetyl-CoA binding. We assign a similar function for His-206 in AgmNAT since its general location in the active site is similar to His-220 in *D. melanogaster* AANATA, and the van der Waals interaction with Pro-35, as described above, is conserved (Fig. 2B). In addition, the His-206 side chain is in van der Waals contact with Ser-168 Cα and Tyr-188 Cε2, as well as several local prolines, Pro-203 and Pro-205. This means that His-206 is contributing to the formation of the active site by interacting with multiple residues. The apo-AgmNAT structure shows Tyr-170 in a position that is not optimal for a direct interaction with His-206 (Fig. 2A), unlike that shown for the corresponding residues in the AANATA structure co-crystalized with acetyl-CoA^15,60^. Tyr-170 occupies space near the entry point for acetyl-CoA into its binding pocket; therefore, we predict that a conformational change will occur that will move Tyr-170 into position for optimal acetyl-CoA binding, possibly by interacting with His-206.

The findings presented in this manuscript highlight mechanistic and structural insights for *D. melanogaster* AgmNAT, an enzyme that catalyzes the formation of *N*-acetylagmatine from acetyl-CoA and agmatine. We provide evidence for an underappreciated reaction in arginine metabolism; however, it still remains unclear if *N*-acetylation of agmatine by an *N*-acetyltransferase enzyme is biologically relevant. A combination of data provided herein and reported from other labs speaks to its relevancy, warranting further investigation into this chemical transformation as a part of arginine metabolism. Furthermore, we outline a chemical mechanism for the AgmNAT-catalyzed formation of *N*-acetylagmatine (and, by extension, other *N*-acylamides), which is consistent with the data presented herein. We also provide evidence for important active site residues involved in substrate binding and maintaining the structural integrity of the active site for efficient catalysis, though further work is necessary to provide more evidence for the dynamic nature of the AgmNAT active site.

## Methods

### Materials

The *AgmNAT* gene was codon optimized and synthesized by Genscript. Ambion RETROscript® Kit, ProBond™ nickel-chelating resin, and MicroPoly(A) Purist^TM^ was purchased from Invitrogen. Oligonucleotides were purchased from Eurofins MWG Operon. PfuUltra High-Fidelity DNA polymerase was purchased from Agilent. BL21 (DE3) *E.coli* cells and *pET-28a*(+) vector were purchased from Novagen. *NdeI*, *XhoI*, Antarctic Phosphatase, and T4 DNA ligase were purchased from New England Biolabs. Kanamycin monosulfate and IPTG were purchased from Gold Biotechnology. Acyl-CoAs were purchased from Sigma-Aldrich. Cayman Chemical commercially synthesized *N* ^1^-acetylspermidine. All other reagents were of the highest quality and purchased from either Sigma-Aldrich or Fisher Scientific.

### AgmNAT: sub-cloning, expression, and purification

*AgmNAT* was inserted into a *pET-28a* vector using *NdeI* and *XhoI* restriction sites, yielding the final expression vector: *AgmNAT-pET-28a*, that after transformation into *E.coli* BL21 (DE3) cells expressed a protein with an N-terminal His_6_-tag followed by a thrombin cleavage site. The *E. coli* BL21 (DE3) cells containing the *AgmNAT-pET-28a* vector was cultured using LB media supplemented with 40 μg/mL kanamycin at 37 °C. The culture was induced with 1.0 mM isopropyl β-D-1-thiogalactopyranoside (IPTG) at an OD_600_ ~ 0.6, followed by an additional four hours at 37 °C. The final culture was harvested by centrifugation at 5,000 × g for 10 min at 4 °C and the pellet was collected. The pellet was resuspended in 20 mM Tris, pH 7.9, 500 mM NaCl, 5 mM imidazole, lysed by sonication, and then centrifuged at 10,000 × g for 15 min at 4 °C. The supernatant was collected and loaded onto 6 mL of ProBond™ nickel-chelating resin, followed by two wash steps: wash one – 10 column volumes of 20 mM Tris, pH 7.9, 500 mM NaCl, 5 mM imidazole followed by wash two – 10 column volumes of 20 mM Tris, pH 7.9, 500 mM NaCl, 60 mM imidazole. AgmNAT was eluted in 1 mL fractions using 20 mM Tris, pH 7.9, 500 mM NaCl, 500 mM imidazole, the protein pooled, and extensively dialyzed at 4 °C against 20 mM Tris pH 7.4, 200 mM NaCl. The concentration of AgmNAT was determined using the Bradford assay indexed against BSA as a standard, and purity was assessed by a SDS-PAGE gel (proteins visualized using by Coomassie stain). Purification of recombinant AgmNAT by nickel affinity chromatography yielded pure protein (≥95%) as visualized by SDS-PAGE (Supplementary Fig. S6).

### AgmNAT crystallography

After nickel-affinity purification, 30 mg of AgmNAT was subjected to dialysis against 50 mM HEPES pH 8.2, 200 mM NaCl, followed by removal of the His_6_ affinity-tag using 60 U of biotinylated thrombin for 18 h in a fresh batch of 50 mM HEPES pH 8.2, 200 mM NaCl leaving an unnatural Gly-Ser-His at the N-terminus. The protein mixture was again subjected to nickel-affinity chromatography to remove undigested AgmNAT. AgmNAT was eluted in the 20 mM Tris, pH 7.9, 500 mM NaCl, 60 mM imidazole fraction, whereas the His_6_-AgmNAT was retained on the column until eluted with 20 mM Tris, pH 7.9, 500 mM NaCl, 500 mM imidazole. The biotinylated thrombin was removed by using 3 mL of Pierce monomeric avidin agarose resin at 4 °C for 30 min, followed by centrifugation to recover AgmNAT, and AgmNAT concentrated to ~10 mg/mL by ultrafiltration. Further purification was performed using a HiTrap Q FF column with a linear gradient from 50 mM HEPES pH 8.2 to 50 mM HEPES pH 8.2, 0.5 M NaCl with AgmNAT eluting in fractions containing ~150 mM NaCl. A final SEC purification step was used after the ion exchange step and purified AgmNAT was concentrated to ~8 mg/ml in 50 mM HEPES pH 8.2, 100 mM NaCl for crystallization screening. The Phoenix crystallization robot and Qiagen screening kits were used to evaluate different crystallization conditions for AgmNAT. AgmNAT was crystallized using the hanging-drop vapor diffusion method in 100 mM Tris pH 8.0, 200 mM sodium acetate, 30% PEG 4000. The drop contained a 1:1 ratio of 1 μL of 8 mg/mL AgmNAT with 1 μL of well solution and incubated at 20 °C. Crystals were of elongated rod-shape. Diffraction was measured at the 22-ID-D SER-CAT beamline at the Advanced Photon Source (APS), Argonne, IL. Data were indexed, scaled, and merged with iMosflm using the CCP4 suite^80^. A homology model was constructed based on the AgmNAT sequence using the program SWISS-MODEL^81^ with mosquito arylalkylamine *N*-acetyltransferase (PDB ID 4FD4)^21^ as a template for molecular replacement. The molecular replacement program Phaser-MR was used in PHENIX. The models of refinement were first obtained using a rigid-body refinement using phenix.refine in PHENIX. PHENIX^82^ and Coot^83^ were used to complete the model rebuilding and refinement. For refinement, data was cut at 2.3 A due to relatively poor data quality at higher resolutions. The crystal structure has been deposited into the Protein Data Bank with accession code 5K9N.

### Construction of AgmNAT site-directed mutants

Site-directed mutants of AgmNAT were constructed by the overlap extension method. Using the primers shown in Table S1, each mutant was amplified using *pfu*Ultra High-Fidelity DNA polymerase with the following PCR conditions: initial denaturing step of 95 °C for 2 min, then 30 cycles of 95 °C for 30 s; 60 °C annealing temperature for 30 s; 72 °C extension step for 1 min; then a final extension step of 72 °C for 10 min. Following the amplification of the *AgmNAT* site-directed mutant, the sub-cloning, expression, and purification procedures are the same as discussed for the wild-type enzyme.

### Measurement of enzyme activity

Steady-state kinetic constants for AgmNAT were determined by measuring the rate of coenzyme A release using Ellman’s reagent (DTNB) at 412 nm (molar absorptivity = 13,600 M^−1^ cm^−1^)^15–17^. The assay consisted of 300 mM Tris pH 8.5, 150 μM DTNB, and the desired concentration of acyl-CoA and amine substrates. Initial velocities were measured using a Cary 300 Bio UV-Visible spectrophotometer at 22 °C. Acyl-CoA kinetic constants were evaluated by holding the concentration of agmatine at a constant saturating concentration (5 mM). Amine kinetic constants were evaluated by holding the concentration of acetyl-CoA at a constant saturating concentration (500 μM). The apparent kinetic constants were determined by fitting the resulting data to equation 1 using SigmaPlot 12.0, where v_o_ is the initial velocity, V_max,app_ is the apparent maximal velocity, [S] is the substrate concentration, and K_m,app_ is the apparent Michaelis constant. Each assay was performed in triplicate and the uncertainty for the k_cat,app_ and (k_cat_/K_m_)_app_ values were calculated using equation 2, where σ is the standard error.

1 $${{\rm{v}}}_{{\rm{o}}}=\frac{{{\rm{V}}}_{{\rm{\max }},{\rm{app}}}[{\rm{S}}]}{{{\rm{K}}}_{{\rm{m}},{\rm{app}}}+[{\rm{S}}]}$$

2 $${\rm{\sigma }}(\frac{{\rm{x}}}{{\rm{y}}})=\frac{{\rm{x}}}{{\rm{y}}}\sqrt{{(\frac{{{\rm{\sigma }}}_{{\rm{x}}}}{{\rm{x}}})}^{2}+{(\frac{{{\rm{\sigma }}}_{{\rm{y}}}}{{\rm{y}}})}^{2}}$$

### Kinetic mechanism and inhibitor analysis

Defining the kinetic mechanism of AgmNAT was accomplished by evaluating double reciprocal plots of initial velocity data for acetyl-CoA and agmatine, followed by determining the type of inhibition for substrate analogs used as dead-end inhibitors or *N*-acetylagmatine for product inhibition. Initial velocities were determined by varying the concentration of one substrate, while holding the other substrate at a fixed concentration. Acetyl-CoA was evaluated at 20, 50, 100, 250 and 500 μM, whereas agmatine was evaluated at 60, 300, 750 and 1500 μM. The resulting initial velocity data was fit to equation 3 for an ordered Bi-Bi mechanism and equation 4 for a ping pong mechanism using IGOR Pro 6.34 A, where v_o_ is the initial velocity, V_max_ is the maximal velocity, K_ia_ is the dissociation constant for substrate A, K_b_ is the Michaelis constant for substrate B, K_a_ is the Michaelis constant for substrate A, [A] is the concentration of substrate A, and [B] is the concentration of substrate B.

3 $${{\rm{v}}}_{{\rm{o}}}=\frac{{{\rm{V}}}_{{\rm{\max }}}[{\rm{A}}][{\rm{B}}]}{{{\rm{K}}}_{{\rm{ia}}}{{\rm{K}}}_{{\rm{b}}}+{{\rm{K}}}_{{\rm{a}}}[{\rm{B}}]+{{\rm{K}}}_{{\rm{b}}}[{\rm{A}}]+[{\rm{A}}][{\rm{B}}]}$$

4 $${{\rm{v}}}_{{\rm{o}}}=\frac{{{\rm{V}}}_{{\rm{\max }}}[{\rm{A}}][{\rm{B}}]}{{{\rm{K}}}_{{\rm{a}}}[{\rm{B}}]+{{\rm{K}}}_{{\rm{b}}}[{\rm{A}}]+[{\rm{A}}][{\rm{B}}]}$$

Inhibition experiments by either substrate analogs or *N*-acetylagamatine were used to discriminate between an ordered, random sequential, or ping pong kinetic mechanism. Oleoyl-CoA, arcaine, and L-arginine methyl ester were used as dead-end inhibitors for AgmNAT while *N*-acetylagmatine was used for product inhibition. Initial velocity patterns were generated by varying the concentration of one substrate, holding the other substrate concentration at its apparent K_m_, and changing the concentration of inhibitor for each data set in triplicate. The resulting data was fit to equations 5–7, for competitive, noncompetitive, and uncompetitive inhibition respectively using SigmaPlot 12.0. For equations 4–6, v_o_ is the initial velocity, V_max,app_ is the apparent maximal velocity, K_m,app_ is the apparent Michaelis constant, [S] is the substrate concentration, [I] is the inhibitor concentration, and K_i_ is the inhibition constant.

5 $${{\rm{v}}}_{{\rm{o}}}=\frac{{{\rm{V}}}_{{\rm{\max }},{\rm{app}}}[{\rm{S}}]}{{{\rm{K}}}_{{\rm{m}},{\rm{app}}}(1+\frac{[{\rm{I}}]}{{{\rm{K}}}_{{\rm{i}}}})+[{\rm{S}}]}$$

6 $${{\rm{v}}}_{{\rm{o}}}=\frac{{{\rm{V}}}_{{\rm{\max }},{\rm{app}}}[{\rm{S}}]}{{{\rm{K}}}_{{\rm{m}},{\rm{app}}}(1+\frac{[{\rm{I}}]}{{{\rm{K}}}_{{\rm{i}}}})+[{\rm{S}}](1+\frac{[{\rm{I}}]}{{{\rm{K}}}_{{\rm{i}}}})}$$

7 $${{\rm{v}}}_{{\rm{o}}}=\frac{{{\rm{V}}}_{{\rm{\max }},{\rm{app}}}[{\rm{S}}]}{{{\rm{K}}}_{{\rm{m}},{\rm{app}}}+[{\rm{S}}](1+\frac{[{\rm{I}}]}{{{\rm{K}}}_{{\rm{i}}}})}$$

### Rate versus pH

The pH-dependence on the kinetic constants for acetyl-CoA was determined using intervals of 0.5 pH units, ranging from 6.5–9.5. Buffers used to measure the pH-dependence were MES (pH 6.5 and 7.0), Tris (pH 7.0–9.0), AmeP (pH 9.0 and 9.5). The resulting data were fit to equations 8 (log (k_cat_/K_m_)_app – acetyl-CoA_ and equation 9 (log k_cat,app – acetyl-CoA_) to determine the apparent pK_a_ values using IGOR Pro 6.34 A, where c is the pH-independent plateau. The wild-type enzyme is reported in triplicate, whereas the E34A mutant was evaluated in duplicate.

9 $$\mathrm{log}\,{(\frac{{{\rm{k}}}_{{\rm{cat}}}}{{{\rm{K}}}_{{\rm{m}}}})}_{{\rm{app}}}=\,\mathrm{log}[{\rm{c}}/(1+{10}^{{{\rm{pK}}}_{{\rm{a}}}-{\rm{pH}}})]$$

10 $$\mathrm{log}\,{{\rm{k}}}_{{\rm{cat}},{\rm{app}}}=\,\mathrm{log}[{\rm{c}}/(1+{10}^{{{\rm{pK}}}_{{\rm{a}}}-{\rm{pH}}})]$$

### Synthesis of N-acetylagmatine

We synthesized *N*-acetylagmatine from putrescine, first converting putrescine to agmatine.

#### Agmatine

To a solution of putrescine (2.0 g, 22.7 mmol) in water (20 mL) was added 2-methylisouronium sulfate (2.7 g, 11 mmol). The mixture was heated to 50 °C for 6 hours, then cooled in an ice bath for 30 minutes. During this time, a white precipitate was formed, which was collected by filtration, and then washed with ice water to give agmatine (1.3 g, 44%) as a white solid that was used without further purification.

^1^H NMR (500 MHz, D_2_O) δ 3.08 (t, *J* = 6.0 Hz, 2 H), 2.81 (t, *J* = 6.8 Hz, 2 H), 1.53 (br. s., 4 H) ppm.^13^C NMR (126 MHz) δ 156.7, 40.5, 39.1, 25.0, 24.7 ppm. LRMS (ESI) m/z: [M + H]^+^ 131.1. HRMS m/z: [M + H]^+^ Calculated for C_5_H_15_N_4_ 131.1297; Found 131.1290.

#### *N*-Acetylagmatine

To a mixture of agmatine (1.0 g, 7.62 mmol) in pyridine (10 mL) was added acetyl chloride (542 μL, 7.62 mmol) dropwise. The mixture was allowed to stir at room temperature for 4 hours, then was concentrated on a rotary evaporator. The crude residue was adsorbed onto silica gel and purified by flash column chromatography (methylene chloride/methanol 19:1) to give *N*-acetylagmatine (400 mg, 30%) as a viscous, colorless oil.

^1^H NMR (500 MHz, CD_3_OD) δ 3.15–3.19 (t, *J* = 6.70 Hz, 2 H), 3.16–3.20 (t, *J* = 6.78 Hz, 2 H) 1.94 (s, 3 H), 1.52–1.64 (m, 4 H) ppm.^13^C NMR (126 MHz) δ 172.0, 157.7, 40.7, 38.3, 26.2, 25.7, 21.1 ppm. LRMS (ESI) m/z: [M + H]^+^ 172.9. HRMS m/z: [M + H]^+^ Calculated for C_5_H_15_N_4_ 173.1402; Found 173.1402.

## Electronic supplementary material

Supplementary Materials
