# Supplementary material for: Structural and Mechanistic Analysis of Drosophila melanogaster Agmatine N-Acetyltransferase, an Enzyme that Catalyzes the Formation of N-Acetylagmatine
Source: Sci Rep. 2017 Oct 18;7:13432. doi: 10.1038/s41598-017-13669-6 (PMC5647378; doi:10.1038/s41598-017-13669-6)
Supplement: Supplementary file 1 — Supplementary Materials [file 41598_2017_13669_MOESM1_ESM.doc]

# Structural and Mechanistic Analysis of *Drosophila melanogaster* Agmatine *N*‑Acetyltransferase,

# an enzyme that Catalyzes the Formation of *N*‑Acetylagmatine

Authors: Daniel R. Dempsey,^1,4,+^ Derek A. Nichols,^2,5,+^ Matthew R. Battistini,^1,+^ Orville Pemberton,^2^
Santiago Rodriguez Ospina,^1^ Xiujun Zhang,^2^ Anne-Marie Carpenter,^1,6^ Brian G. O’Flynn,^1^

James W. Leahy,^1,2,,3^ Ankush Kanwar^1^, Eric M. Lewandowski,^2^ Yu Chen,^2*^ and David J. Merkler^1**^

^+^These authors contributed equally to this work.

^1^Department of Chemistry, University of South Florida, Tampa, Florida 33620

^2^Department of Molecular Medicine, University of South Florida, Tampa, Florida 33612

^3^Florida Center of Excellence for Drug Discovery and Innovation, 3720 Spectrum Boulevard, Suite 305, Tampa, FL 33612

^4^Current Address: Johns Hopkins University, School of Medicine, Baltimore, MD 21205

^5^Current Address: Moffitt Cancer Center, Tampa, FL 33612

^6^Current Address: University of Florida, College of Medicine, Gainesville, FL 32610-0216

Table S1.

Site‑directed Mutagenesis Primers

| Mutant | Forward or Reverse Primer | Primer Sequence |
| --- | --- | --- |
| E34A | Forward | TTA TTA CCC GGA AGC ACC GCT GAC CGC CG |
|  | Reverse | CGG CGG TCA GCG GTG CTT CCG GGT AAT AA |
| P35A | Forward | CGC ATT ATT ACC CGG AAG AAG CGC TGA CCG CC |
|  | Reverse | GGC GGT CAG CGC TTC TTC CGG GTA ATA ATG CG |
| S171A | Forward | AAC CAG ACG CGC GGC GTA CAC ACT CGT AC |
|  | Reverse | GTA CGA GTG TGT ACG CCG CGC GTC TGG TT |
| H206A | Forward | TCC GCC GCC GCC GGC TGA ATC GGT CCA A |
|  | Reverse | TTG GAC CGA TTC AGC CGG CGG CGG CGG |


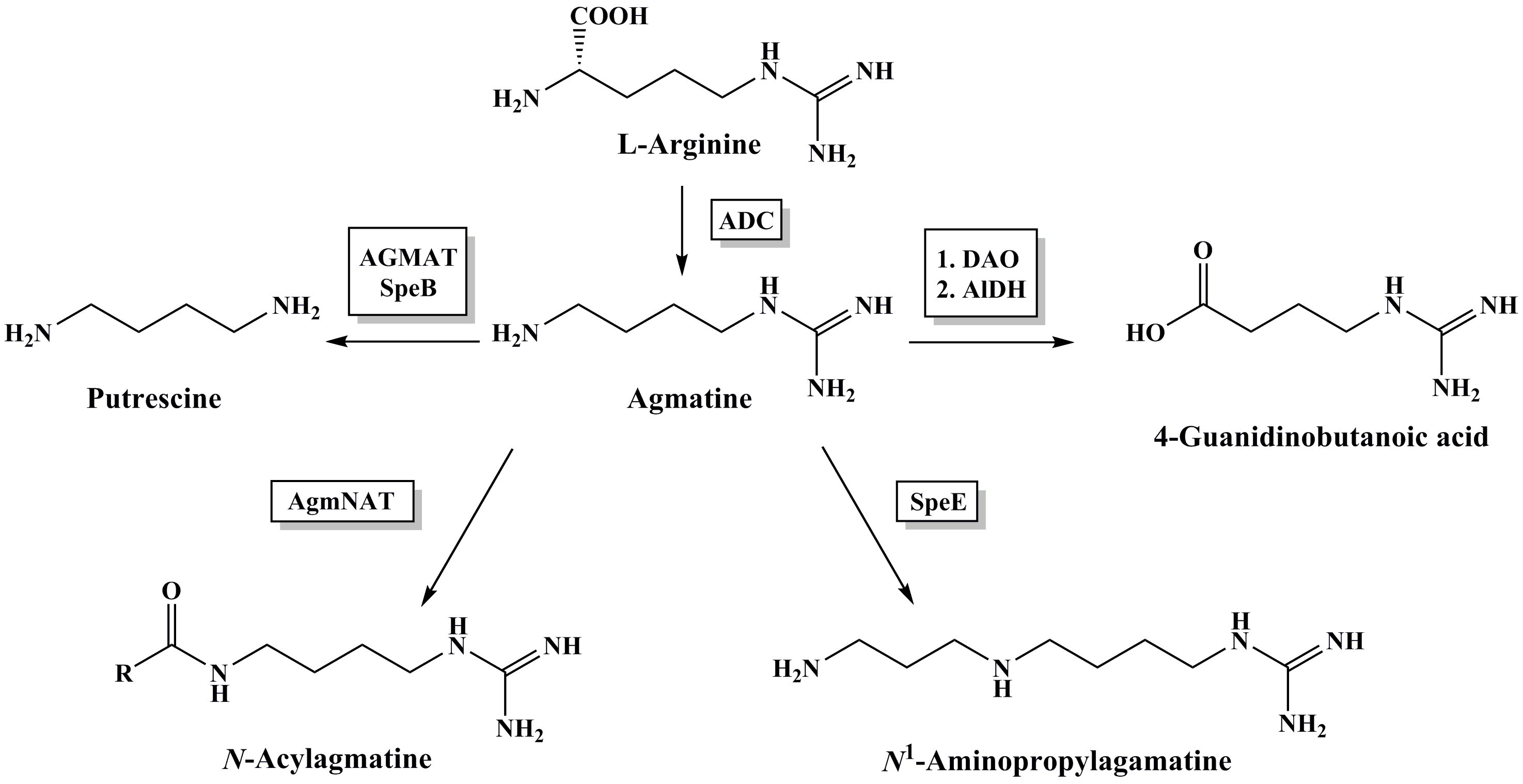


**Figure S1.** Agmatine metabolism. arginine decarboxylase (ADC), agmatinase (AGMAT), diamine oxidase (DAO), aldehyde dehydrogenase (AlDH), agmatine *N*‑acetyltransferase (AgmNAT), and polyamine aminopropyltransferase (SpeE).


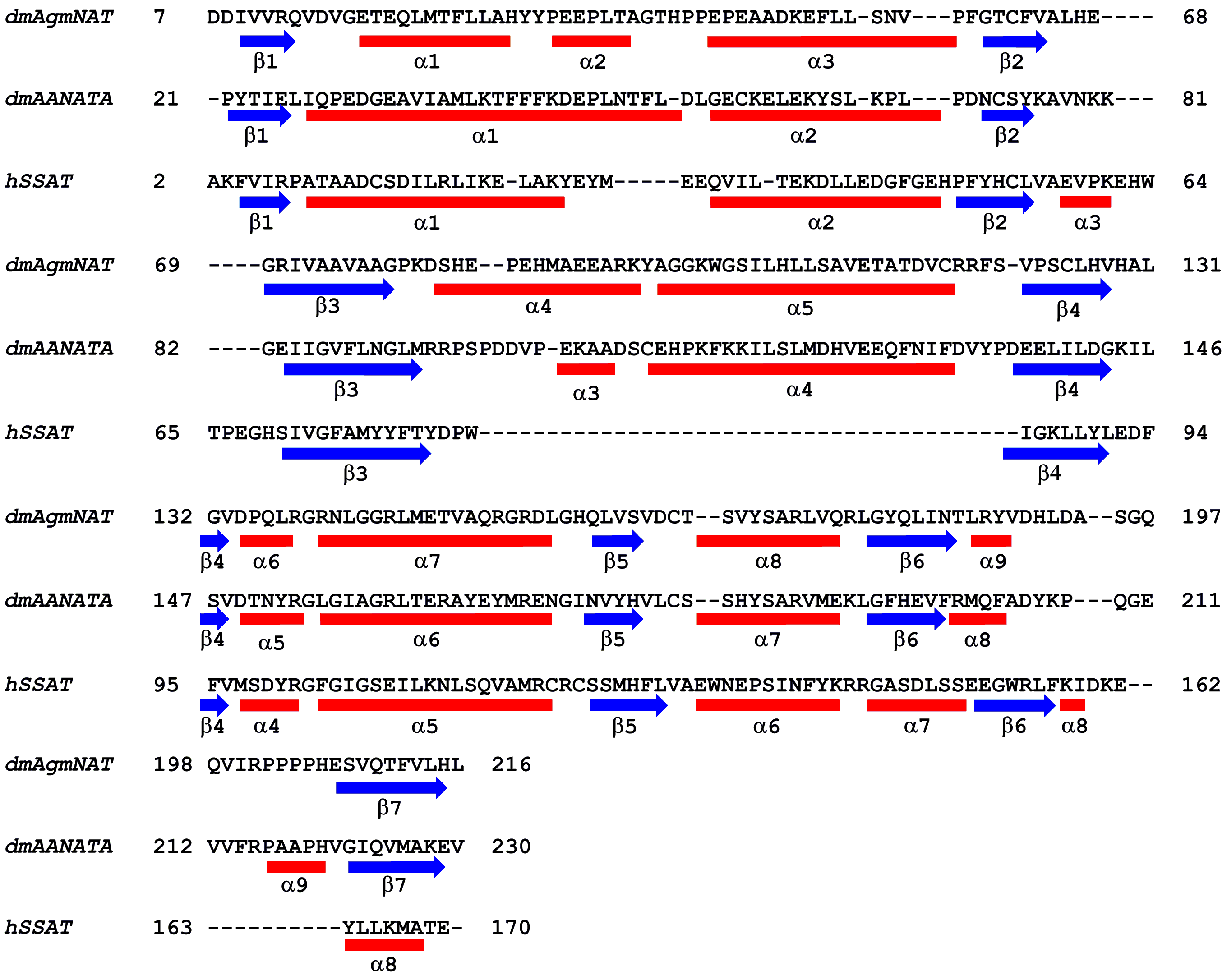


**Figure S2.** Multiple sequence alignment of AgmNAT with *D. melanogaster* AANATA and human SSAT.


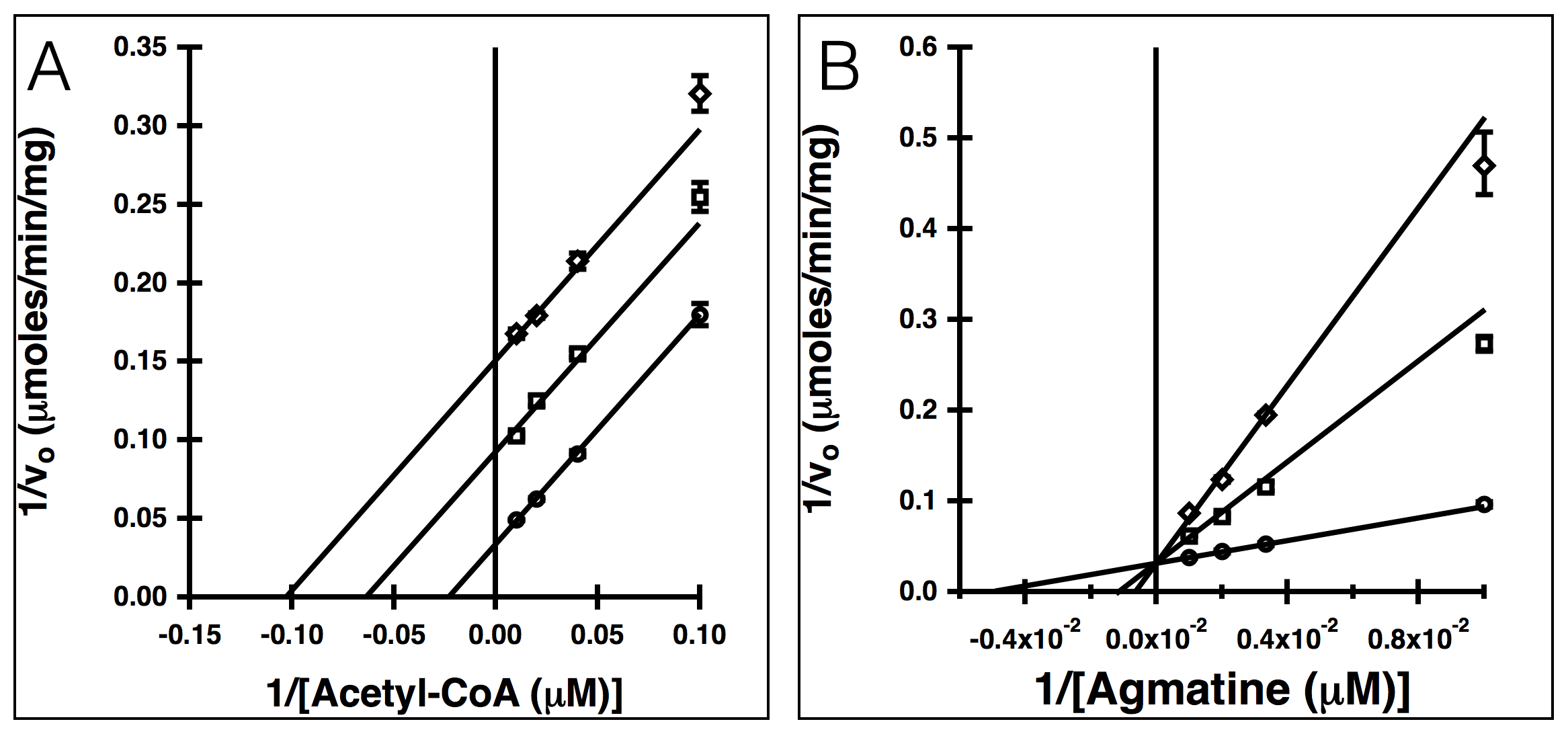


**Figure S3.** AgmNAT – arginine methyl ester inhibition analysis. (A) Initial velocities measured at a fixed concentration of agmatine (300 μM), varying the concentrations of acetyl‑CoA, and varying the concentrations of the inhibitor, arginine methyl ester: 0 nM (○), 5 mM (🞏), 10 mM (**◇**); K_i_ = 2.9 ± 0.1 mM. (B) Initial velocities measured at a fixed concentration of acetyl‑CoA (104 µM), varying the concentrations of agmatine, and varying the concentrations of the inhibitor, arginine methyl ester: 0 nM (○), 5 mM (🞏), 10 mM (**◇**); K_i_ = 1.5 ± 0.1 mM.


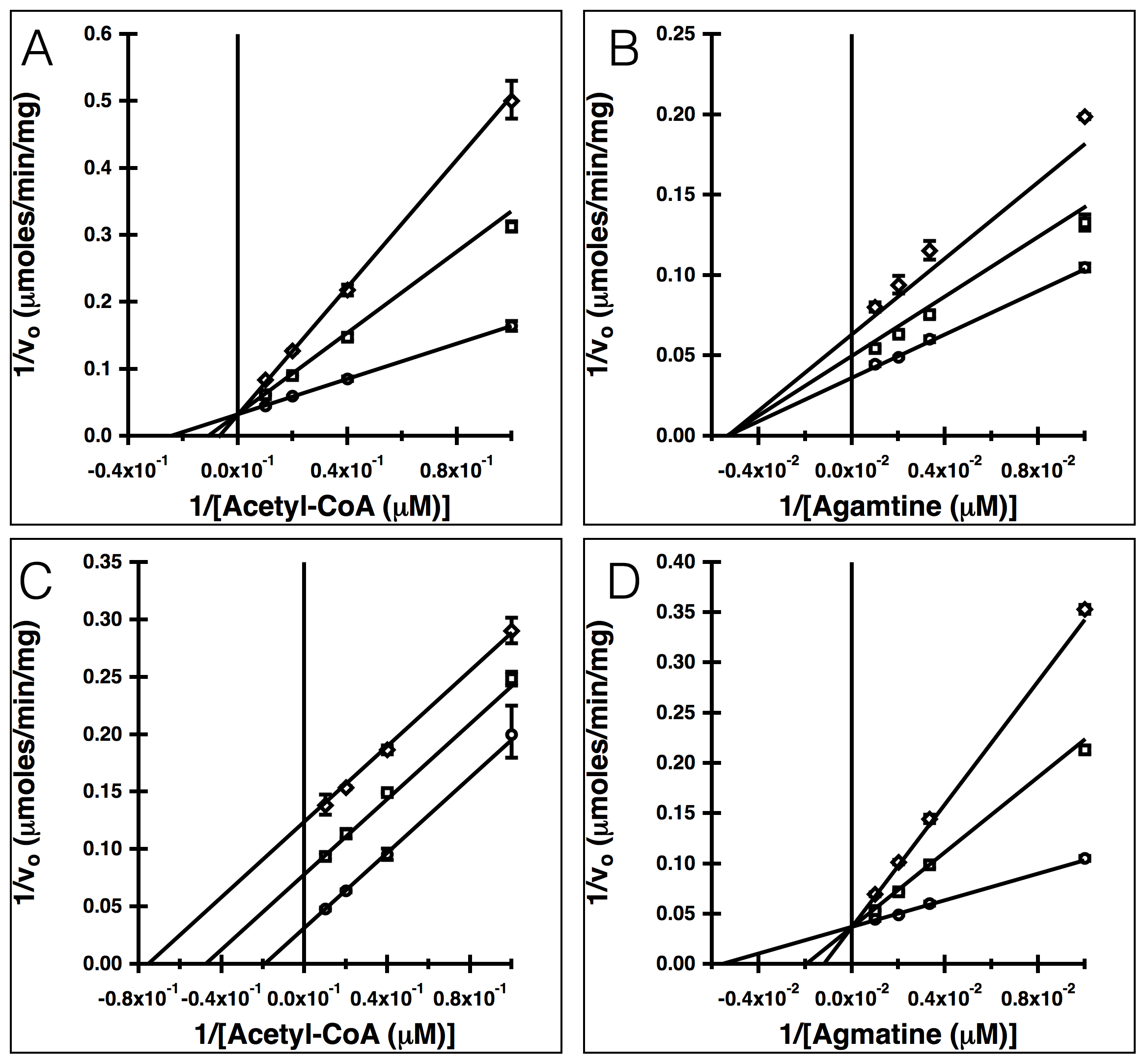


**Figure S4.** Dead-end inhibition analysis of AgmNAT. (A) Initial velocities measured at a fixed concentration of agmatine (300 µM), varying the concentrations of acetyl‑CoA, and varying the concentration of the inhibitor, oleoyl‑CoA: 0 μM (○), 25 μM (🞏), 50 μM (**◇**); K_i,s_ = 19 ± 1 μM. (B) Initial velocities measured at a fixed concentration of acetyl‑CoA (104 µM), varying the concentrations of agmatine, and varying the concentration of the inhibitor, oleoyl‑CoA: 0 μM (○), 25 μM (🞏), 50 μM (**◇**); K_i,s_ = K_i,i_ = 67 ± 4 μM. (C) Initial velocities measured at a fixed concentrations of agmatine (300 μM), varying the concentration of acetyl‑CoA, and varying the concentration of the inhibitor, arcaine: 0 μM (○), 50 μM (🞏), 100 μM (**◇**); K_i,i_ = 34 ± 1 μM.
(D) Initial velocities measured at a fixed concentration of acetyl‑CoA (104 μM), varying the concentrations of agmatine, and varying the concentration of the inhibitor, arcaine: 0 μM (○),
50 μM (🞏), 100 μM (**◇**); K_i,s_ = 28 ± 1 μM.

| **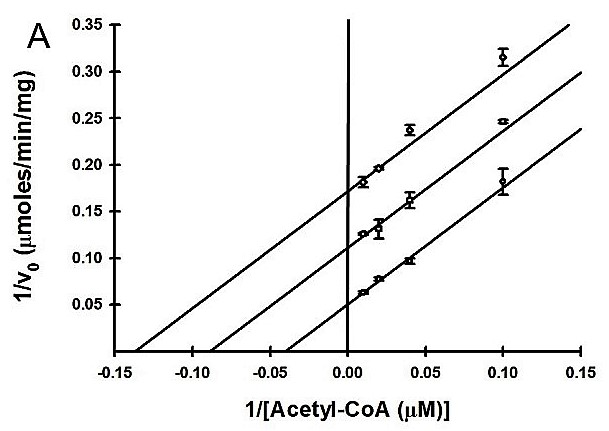** | **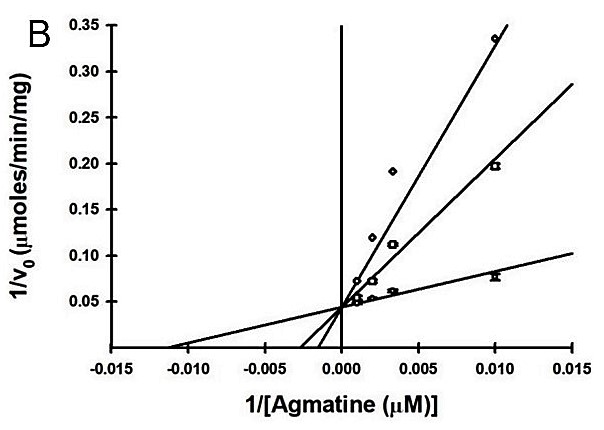** |
| --- | --- |

**Figure S5.**  Product inhibition of AgmNAT by *N*-acetylagmatine.  (A) Initial velocities measured at a fixed concentration of agmatine (300 μM), varying the concentrations of acetyl‑CoA, and varying the concentration of the inhibitor, *N*-acetylagmatine: 0 mM (○), 0.5 mM (🞏), 1.0 mM (**◇**); K_i,i_ = 420 ± 20 μM.  (B) Initial velocities measured at a fixed concentration of acetyl‑CoA (104 μM), varying the concentrations of agmatine, and varying the concentration of the inhibitor, *N*-acetylagmatine: 0 mM (○), 0.5 mM (🞏), 1.0 mM (**◇**); K_i,s_ 160 ± 30 μM.


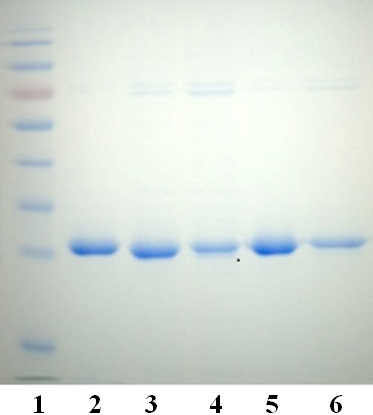


**Figure S6.** AgmNAT and mutant purification. Lane 1 - PageRuler™ Prestained protein ladder
(Thermo Scientific), Lane 2 – wild-type, Lane 3 – E34A, Lane 4 – P35A, Lane 5 – S171A, Lane 6 – H206A.
